# Investigating the Nursing Practitioners Perspectives about Undergraduate Nursing Internship and Apprenticeship Courses: Is Renewing Required?

**DOI:** 10.5539/gjhs.v5n5p144

**Published:** 2013-06-23

**Authors:** Ali Jamalmohammadi, Mohammad Asghari Jafarabadi, Jila Shajari, Maryam Modares

**Affiliations:** 1 Department of Medical Education, Virtual School, Center of Excellence for E-learning in Medical Education, Tehran University of Medical Sciences, Tehran, Iran; 2 Medical Education Research Center, Faculty of Health, Tabriz University of Medical Sciences, Tabriz, Iran; 3 Faculty of Nursing and Midwifery, Tehran University of Medical Sciences, Tehran, Iran

**Keywords:** nursing practitioners, training courses, syllabus

## Abstract

Nurses’ professional capacity plays an important role in the health system to achieve their mission. This study aimed to investigate the perspectives of nursing practitioners about undergraduate nursing internship and apprenticeship courses and possible ways of renewing the courses.

This cross sectional survey was performed over 258 bachelors and practitioners of nursing graduates of Alborz University of medical sciences in the second half of 2012. Based on a multi-stage sampling schedule, questionnaires were used to collect data about the perspectives of nursing practitioners about undergraduate nursing internship and apprenticeship courses.

There were 81.4% of females and 80.6%, 17.1% and 2.3% of organizational post of participants were nurse, head nurse and supervisor respectively. The occupied posts for 60.1%, 25.6% and 14.1% of subjects, respectively were nurse, head nurse and the supervisor. The application of the internship and apprenticeship courses in bachelor of nursing were in moderate to high levels. The highest percentages of responses for internship and apprenticeship training courses were in internal surgery nursing and special nursing and the minimum percentage of responses were for community hygiene nursing and mental health nursing.

Due to observing moderate to high levels of fulfillment and lack of compliance of training courses, renewing to improve the quality and effectiveness of training programs are highly recommended. This can be effective in the future of nursing career and provide a practical training environment to achieve the goals of theoretical training and can lead nurses to become specialized in their field.

## 1. Introduction

Accuracy and quality of education to meet the needs of the employers and their job requirements are very important and necessary (Fasihi harandi, Soltani arabshahi, Tahami, & Mohammadalizadeh, 2004). The compliance of training courses with job requirements would be more evident in the field of medical science because the presence of qualified personnel, particularly personnel with sufficient knowledge has an important role to improve the quality of health care (Harvabadi & Marbaghi, 1996). After a decade of motion to promote the quantity of education in the field of medical sciences (Khorgami, Danaie, & Damari, 2002), it is more than two decades that medical centers in the world are concerned about the efficiency of that (Shahhosseini, 1998). Amongst, the nursing has a major contribution, since as the members of health teams, nursing graduates are contributed in different areas of health care, education, research, counseling, prevention, management and support, medical care and rehabilitation (Classification and job evaluation schemes of ministries, 2003). The mission of undergraduate nursing education is to train a knowledgeable workforce, committed, skilled and efficient, with professional skills in the field of health care and rehabilitation by meeting the highest standards, to offer maintaining and improving public health. This can be achieved through continuous education, research and development of nursing knowledge.

Various studies investigated the limitations of traditional nursing education program and functional impairment of theoretical and practical training methods based on traditional and classical design and the effect of new learning method on students’ clinical competence (Minaee, 2010). In our country, up to now, studies have been done to examine the compliance of the contents of courses with the work requirements in other medical disciplines which showed the inefficiency of theoretical trainings concerning their professional needs and they recommended the renewing of education program (MohammadPour & Matlabi, 2002; Hosseini & Sarchami, 2005; Ghazanfari et al., 2010).

However, based on vast searching in the literature, there was no study, if any, about the compliance of the internship and apprenticeship training courses of Bachelor of Science (B.Sc.) of nursing with their educational needs to enable them for performing their professional tasks. With attention to the importance of responsibility and activity of B.Sc. of nursing in healthcare positions and their effective role, studies in this area is highly necessary. Considering the necessity of achieving the goals of clinical skills in nursing, it seems the content of internship and apprenticeship training courses can provide the professional needs of graduates in this field. With regard to above mentioned rationales, this study aimed to investigate the perspectives of nursing practitioners of the Alborz University of medical sciences about undergraduate nursing internship and apprenticeship courses.

## 2. Methods

This cross-sectional survey was conducted in the population of nurses working in hospitals in all subsets of Alborz University of medical sciences, Alborz Province, Karaj, Iran, in the second half of 2012. For sample size determination, the information about the primary outcome of study i.e. the percentage of compliance of internship and apprenticeship training courses with professional needs was obtained based on study done by Ghazanfari et al. (2009). Taking into account the 95% confidence and using the formula for determining the sample size for proportions in descriptive studies, a total of 258 cases were computed to recruit in the study.

### 2.1 Study Participants

Considering the organizational chart and guidelines related to human resources of the ministry of health and medical education, all organizational posts that meet the conditions of nursing employment and could be converted, were extracted. Then a list of responsibilities and duties defined by the reform committee on medical administrative commission of institutional structures was collected and was compiled for each organizational posts (Classification and job evaluation schemes of ministries, 2003). A multi-stage sampling was used to select 10 hospitals in the first stage and then proportionately to select nurses within each hospital by simple random sampling schedule within each hospital.

To have a B.Sc. degree in nursing that entered in college since 2005 (to assess the opinion of recent cohort of nursing students), has working in the health system and willing to participate in the study were the inclusion criteria. Subjects, who didn’t will to participate in the study, didn’t completely answer the questionnaire or transferred to outside areas of Alborz University of medical sciences, were excluded from the study.

### 2.2 Data Collection

A researcher designed questionnaire was used to collect data. The content validity was evaluated by a panel of 35 experts in the field of medical education and nursing.After some minor revisions the validity were confirmed. Internal consistency reliability of the questionnaire was assessed and confirmed using Cronbach's alpha coefficient for the two sets of closed questions related to assess the adequacy of training courses of internship (α=.89) and apprenticeship (α=.91). The questionnaire consisted of two parts: in the first part, a series of questions were asked about personal characteristics including gender, entry date to undergraduate courses, leaving dates of the period, working starting date, as well as organizational and occupied posts. In the second part, the questionnaire consisted of scales of applicability of courses involving 1) closed questions regarding the educational curriculum offered internship and apprenticeship training courses in the line with their professional duties as well as 2) open questions regarding suggestion for changing in courses and curricula. Participants answered the applicability of each course regarding their job duties in each closed item by very high (ranked by 5), high (4), moderate (3), low (2) and very low (1). In the introduction section of questionnaire it was explained how to answer the questions. The questionnaire was completed self descriptively which takes about 5 to 10 minutes.

### 2.3 Ethical Consideration

This study was authorized by the ethics committee of Alborz University of Medical Sciences. In order to study meet the medical ethics, the information was completely private and was unrelated to the personal information. In addition, a written consent was completed by each participant before completing the questionnaire.

### 2.4 Statistical Analysis

Data were summarized by frequency (percentage). Prioritization and comparison of the internship and apprenticeship training courses with regard to its application in their professional clinical capabilities were done using the Friedman test and in the case of significant results of this test, for pair wise comparisons post hoc tests with Bonferoni correction were used. Also the agreement between organizational posts and occupied posts was assessed by Cohen's Kappa. Values < 0.4, 0.4-0.7 and > 0.7 respectively indicate the weak, moderate and strong agreement. All analyses were done using SPSS13 software (SPSS Inc, IL, Chicago, USA) at the significance level of 0.05.

## 3. Results

There were a number of 48 males (18.6%) and 210 females (81.4%) in the study. The 80.6%, 17.1% and 2.3% of organizational post of participants were nurse, head nurse and supervisor respectively. The occupied posts for 60.1%, 25.6% and 14.1% of subjects, respectively were nurse, head nurse and the supervisor (Table 1). There was a significant and moderate agreement between organizational posts and occupied posts (Cohen's Kappa=.47, P < 0.001), so that a total of 194 persons (75.2%) were correctly employed as their organizational posts.

**Table 1:** Summary statistics of study participants

Characteristics	N	%
**Gender**		
Female	48	18.6
Male	210	81.4
**Entry Date to undergraduate courses**		
2005	158	61.2
2006	75	29.1
2007	25	9.7
**Leaving dates of the period**		
2009	146	56.6
2010	87	33.7
2011	25	9.7
**Work beginning dates**		
2009	125	48.4
2010	89	34.5
2011	32	12.4
2012	12	4.7
**Organizational posts**		
nurse	208	80.6
head nurse	44	17.1
supervisor	6	2.3
**Occupied posts**		
nurse	155	60.1
head nurse	66	25.6
supervisor	37	14.3

### 3.1 Application of Apprenticeship Training Courses with Professional Clinical Capability

Results of the respondents’ opinions about the level of compliance and relatedness of apprenticeship training courses of undergraduate nursing with their professional clinical capability of participants involved in the study showed that nine syllabus including nursing skills, mothers and neonatal health, community hygiene nursing, internal surgery nursing 1, internal surgery nursing 2, internal surgery nursing 3, internal surgery nursing 4, children nursing and mental health nursing were moderate to high level related to the with their professional clinical capability so that the most of the participants rated “moderate” to “very high” the applicability of the related syllabus (Table 2).

Furthermore, the results of Friedman ranking for the prioritization of undergraduate nursing apprenticeship training courses in terms of their application in clinic showed that the internal surgery nursing 4, internal surgery nursing 3, internal surgery nursing 2, internal surgery nursing 1 and nursing skills, respectively with the highest ranks were in the highest priority, and the syllabus of mothers and infants health and children nursing by acquiring the next lower ranks, respectively were in the second-level priority, mental health nursing by acquiring the next lower rank was in the third-level priority, and finally the syllabus of community hygiene nursing by getting the lowest rate (Table 2), was in the lowest level of importance in the respondents’ view point (P <0.05 for all comparisons).

**Table 2:** Application of apprenticeship training courses with professional clinical capability

Training courses	Very low		Low		Moderate		High		Very High		Friedman rank

	N	%	N	%	N	%	N	%	N	%
internal surgery nursing 4	0	0	5	1.9	40	15.5	76	29.5	137	53.1	5.84
internal surgery nursing 3	0	0	4	1.6	37	14.3	86	33.3	131	50.8	5.83
internal surgery nursing 2	1	0.4	4	1.6	40	15.5	82	31.8	131	50.8	5.78
internal surgery nursing 1	2	0.8	5	1.9	40	15.5	83	32.2	128	49.6	5.71
nursing Skills	0	0	7	2.7	49	19	93	36	109	42.2	5.36
children nursing	8	3.1	13	5	55	21.3	86	33.3	96	37.2	4.95
mothers and infants health	5	1.9	14	5.4	62	24	86	33.3	91	35.3	4.74
mental health nursing	7	2.7	32	12.4	83	32.2	79	30.6	57	22.1	3.64
community hygiene nursing	11	4.3	37	14.3	104	40.3	64	24.8	42	16.3	3.15

### 3.2 Application of Internship Training Courses with Professional Clinical Capability

Results showed that all twelve syllabus including mothers and neonatal health, community hygiene nursing, internal surgery nursing 1, internal surgery nursing 2, internal surgery nursing 3, internal surgery nursing 4, children nursing1, children nursing2, mental health nursing, especial nursing, the principles of management in nursing services and nursing in crisis, urgency and extraordinary phenomena were significantly related to the professional clinical capability of nurses so that the most of the participants rated “moderate” to “very high” the applicability of the related syllabus (Table 3).

**Table 3:** Application of internship training courses with professional clinical capability

Training courses	Very low		Low		Moderate		High		Very High		Friedman rank

	N	%	N	%	N	%	N	%	N	%
special nursing	2	0.8	9	3.5	35	13.6	73	28.3	139	53.9	7.72
internal surgery nursing 4	0	0	7	2.7	40	15.5	79	30.6	132	51.2	7.55
internal surgery nursing 3	0	0	8	3.1	39	15.1	83	32.2	128	49.6	7.52
internal surgery nursing 2	1	0.4	7	2.7	40	15.5	83	32.2	127	49.2	7.46
internal surgery nursing 1	0	0	6	2.3	46	17.8	85	32.9	121	46.9	7.34
children nursing 1	7	2.7	8	3.1	58	22.5	87	33.7	98	38	6.57
children nursing 2	6	2.3	11	4.3	58	22.5	92	35.7	91	35.3	6.41
mothers and infants health	5	1.9	12	4.7	73	28.3	79	30.6	89	34.5	6.08
nursing in crisis, urgency and extraordinary phenomena	5	1.9	25	9.7	55	21.3	84	32.6	89	34.5	6.01
principles of management in nursing services	6	2.3	17	6.6	72	27.9	80	31	83	32.2	5.85
mental health nursing	3	1.2	19	7.4	88	34.1	81	31.4	67	26	5.38
community hygiene nursing	12	4.7	38	14.7	101	39.1	72	27.9	35	13.6	4.09

Furthermore, the results of Friedman ranking for the prioritization of undergraduate nursing internship training courses in terms of their application in clinic showed that the especial nursing, internal surgery nursing 4, internal surgery nursing 3, internal surgery nursing 2 and internal surgery nursing 1 with the highest ranks were respectively in the highest priority, and the syllabus of children nursing 1, children nursing 2, mothers and infants health and nursing in crisis, urgency and extraordinary phenomena respectively by acquiring the next lower ranks were in the second-level priority, mental health nursing by acquiring the next lower rank was in the third-level priority, and finally the syllabus of community hygiene nursing by getting the lowest rate (Table 3), was in the lowest level of importance in the respondents’ perspectives (P < 0.05 for all comparisons).

### 3.3 Strengths and Shortcomings of Nursing Internship and Apprenticeship Courses

In relation to the high priority of the internship and apprenticeship courses, the participants noted some strength which the major points outlined as follow:

-Dedication of required headings in the curriculum
-Approximate agreement among theoretical with internship and apprenticeship courses
-Practical application of courses regarding caring of their patients
-Involving all areas of treatment and patients
-Extensive and wide applicability of the internal surgery nursing courses both in theory and internship
-Existence of experienced and high qualified teachers in internal surgery nursing courses
-Highly effective using of these lessons had been effective in increasing the knowledge
-Compliance the most of courses in the curriculum with professional capabilities
-Nursing process offered in the courses
-The presence of nurses in the hospital and intensive care unit in accompany with a physician

In relation to the low priority of the internship and apprenticeship courses, the participants noted some shortcoming which the major points outlined as follow:

-Lack of key and practical points which could be applicable in future situations
-Not experiencing the strengths and capabilities of professional duties
-Thick books that are just too expensive
-Students not allowed to take care of the patients and to strengthen their skills and only seeing a few cases which are not enough to enter them into the real world
-Lack of coordination as well as concurrent compliance of theoretical and practical courses which leads in problems while working
-Not being up to date in the lessons that must be used at all working times
-Reduction and short hours of practical courses
-Loosing time in internship program due to lack of qualified coaches and teachers
-Using lecture notes instead of using the current nursing books
-The lengthy nature of the courses
-Not paying enough attention to many of the topics that have been addressed in the curriculum
-Lack of or limited authority in the case of internship courses which make them inefficient in the real situation

### 3.4 Suggestions to Change the Headings of Training Program

There were suggestions to add sonography (5.43%), radiology (5.43%) and medical equipment (32.95%) in nursing internship courses and to add Pathology (8.91), Neonatal intensive care (15.50%), airway management (29.46%), Casting and splint (26.74%), Emergency Nursing (10.85%), Triage (33.33%) and physical examinations (15.50%) in nursing apprenticeship courses.

There were suggestions to eliminate community hygiene nursing (4.26%) in current nursing internship courses and also to eliminate community hygiene nursing (4.26%) in current nursing apprenticeship courses. However there were no suggestions to eliminate other current courses both in internship and apprenticeship courses.

In addition there were suggestions to promote the nursing B.Sc. course program which the major points outlined as follow:

-Devoting more time to practical training courses instead of more than 3 years of theoretical courses because of weakness of fresh graduates in practice
-Simultaneous presenting of the theoretical and practical courses for students in order to not forget the theory while being in practice
-To put into practice the scientific knowledge in apprenticeship programs
-More units to be devoted for internships and apprenticeship courses
-Elimination of community hygiene nursing course due to lack of compliance and applicability in clinical nursing practice
-More practical presentation of special nursing course
-Use of equipment and skilled and experienced clinical educators to teach students in order to learn the clinical experience accompany with the new knowledge
-Review in how to properly promote apprenticeship and practical training courses
-Providing related workshops while working

## 4. Discussion

Based on the perspectives of nursing participants in this study, the application of internship and apprenticeship training courses in B.Sc. of nursing were in moderate to high level. Because the intermediate and higher levels of application of internship and apprenticeship courses cannot be satisfactory and the since there were lack of full compliance of training courses with the job requirements, it is necessary to renew these courses or at least renew their headlines. Especially this is more necessary in the field of nursing field (Harvabadi & Marbaghi, 1996,)

Similar to our study, other studies concluded the lack of full compliance of the training courses regarding the job requirements; a research on medical graduates done by Ghazanfari et al. (2010) concluded that the adaptation of clinical education program is not sufficient for their job requirements. Improving the quality of clinical education is strongly needed to increase the efficiency of education program for medical alumni in their future career. Results of the study done by Hamdi et al. (2005) on environmental health graduates also found lack of compliance of the courses and concluded that to implement training courses for graduates, considering their educational needs and their dominant administrative functions are highly necessary. These amendments may include the updating headlines and content of the courses or designing of more important practical and internship courses (Sabouri, Shayan, & Salehi, 2002). In other study, there was a lower percent of students who were able to perform their skills and the most skills were theoretically trained and also practical educations were presented with insufficient discussion and comment (Yousefi, Pourebrahim, & Sinaee, 2008). However, in the study done by Beigmoradi and Nazeri (2000) in the last year nursing students, they found that in the nursing educational program, the majority of students rated good and higher level the realization of the programs goals. But the other parts of their results suggested that some training units of internships and apprenticeships nursing courses were not sufficient and there was a poor training and students reported their poor capability.

Evaluation of the rate of application of internship and apprenticeship courses of B.Sc. of nursing also showed that the internal surgery nursing and special nursing in internship and apprenticeship courses had the most priority and community hygiene nursing and mental health nursing courses had the least priority in application. This difference may also reflect the lack of full compliance of training courses with job responsibilities and requirements. The participants had suggestions to eliminate community hygiene nursing and mental health nursing courses from both nursing internship and apprenticeship program due to lack of compliance and applicability of these courses in clinical nursing practice. While the priority to remove the declared courses by the nurses employed in the different organizational posts were different, though it was consistent with differences existed in the application of courses in each organizational posts. Therefore the courses presented may be without application or with the least application considering the type of their job. So the elimination or renew of a number of units of internship and apprenticeship courses can be suggested considering nursings’ job requirements. In the line with this finding, Sabouri et al. (2002), about the reorganizing the educational process of medical internship courses, concluded that for effective education, the educational need assessment is essential and it was highly recommended to prioritize the less important courses and educational materials in the next levels.

Nurses also had suggestions for adding some units of internships and apprenticeships courses or adding new units of these courses. An investigation through these suggestions revealed some unfulfilled job requirements of the nurses. A series of the courses did not exist in the education period or have been paid less attention. Suggestions like adding training courses about the familiarity with medical equipment and their application, ultrasound and radiology and pathology, neonatal intensive care, airway management, casting and splinting, emergency care, triage and a physical examination are the needs requisite of their job. Many suggestions were related to adding units of apprenticeships courses. Since the apprenticeships courses are directly in the line with performance tasks, assigned job and their responsibilities, it seems that the suggestions are completely based on their real needs. Among the recommendations regarding to increase the units of courses, there were differences between experts employed in various responsibilities which could be explained by the differences in their job tasks and with attention to specific needs for performing them. Like our finding, in the study by Hamdi et al. (2005), employees of the environmental health suggested adding new educational courses regarding their educational needs considering the functional and executive activities.

Therefore to achieve the promotion and optimal compliance, the clinical training should be so designed that to provide clinical skills for mastering of students, and enable them to use these skills in their job environment (Hunsberger et al., 2000). Since, there was significant relationship between learning method and the program set (Jang, 1993), planning in how to study and making incentives in students for personal studies would strengthen the student's learning rate. Parker et al. (2003) studied the impact of suitable and performance based training program for the adaptation of the clinical roles, and according to traditional methods, they found that students would encounter their new role in the clinic environment without any specific and systematic training program. Also the training program is not able to assess their performance. So incorporating educational program with new ways of teaching which lead to the development of clinical competence in students should be considered and evaluated (Minaee, 2010). Amini et al. (2005) in their study on dentistry students found that students are required to be educated in the most practical skills associated to their jobs. They also concluded that it is necessary to have accurate plans for training of practical clinical skills for these students.

There are some studies suggesting new ways of teaching; as a part of the study done by Dianati and Adib Haj Bagheri (2005), about the educational needs of nurses, it has been announced that conferences and simultaneous training are the best training methods from the perspective of nurses. Results of a similar study indicated those interns who only visit the cases had less ability comparing to those who manage the cases themselves or under a supervisor. In this study, if the learners were exposed to different learning and teaching processes, they would better manage the cases referred (Shams, 2008).

In this regard, the nursing participants in this study also recommendation for devoting more time to practical training courses and to put into practice the scientific knowledge, simultaneous presenting of the theoretical and practical courses, using of equipment and skilled and experienced clinical educators and providing related workshops while working. Based on the results of this research, the managers and education developers should adjusted and or renew their program based on continues evaluation plan for learners to enable them for achieving sufficient scientific benefits (Manuaba, 1997).

There were some limitations in this study; One of limitations of the study is the generalize ability of the results to the whole country, because of small sample size and sampling from just one state. In addition, the adaptation was only assessed based on the perspectives of employed experts of this field. It is better to evaluate the amount of adaptation and the application of educational courses with expected and in progress tasks, based on a set of total available resources. This would include scientific and executive documentation, expert's comments and scientific and technical connoisseurs of the field to offer more valid and confident results in order to reform and making changes.

In conclusion, the study showed that compliance with the requirements of professional nursing education program is in moderate and higher levels and therefore not satisfactory. Additionally, internal surgery nursing and special nursing in internship and apprenticeship courses had the most priority and community hygiene nursing and mental health nursing courses had the least priority in application. Hence, with attention to the results of this study, renewing in a number of units and in the headlines of some nursing internship and apprenticeship courses is suggested. Therefore, further studies are highly recommended under the supervision of the ministry of health and medical education and in line with the experts’ opinions. Also it is emphasized to study the documentation of educational program of the field and task analysis of the bachelors. Then renewing to improve the quality and effectiveness of training programs could be effective in order to increase the compliance based on continues supervising of the masters of the field, improving the relationship between teacher and student, supplying of information resources to provide different levels of learning and upgrading the quality of evaluation methods of interns and apprentices. Providing an effective practical learning environment could be effective to achieve the theoretical learning goals and can lead the nursing practitioner to become specialized in their field. Furthermore for promoting and learning behavior and functional skills of alumni, the internship courses are suggested to begin in beginning of the semesters. Removing of some training courses units with lower levels of application and adding a number of units with higher levels of application and continues updating of headlines and the content of courses is also recommended.
